# A data and text mining pipeline to annotate human mitochondrial variants with functional and clinical information

**DOI:** 10.1002/mgg3.1085

**Published:** 2019-12-10

**Authors:** Ornella Vitale, Roberto Preste, Donato Palmisano, Marcella Attimonelli

**Affiliations:** ^1^ Department of Biosciences, Biotechnologies and Biopharmaceutics University of Bari Bari Italy

**Keywords:** annotation, mitochondria, pathogenicity, variant

## Abstract

**Background:**

Human mitochondrial DNA has an important role in the cellular energy production through oxidative phosphorylation. Therefore, this process may be the cause and have an effect on mitochondrial DNA mutability, functional alteration, and disease onset related to a wide range of different clinical expressions and phenotypes. Although a large part of the observed variations is fixed in a population and hence expected to be benign, the estimation of the degree of the pathogenicity of any possible human mitochondrial DNA variant is clinically pivotal.

**Methods:**

In this scenario, the establishment of standard criteria based on functional studies is required. In this context, a “data and text mining” pipeline is proposed here, developed using the programming language R, capable of extracting information regarding mitochondrial DNA functional studies and related clinical assessments from the literature, thus improving the annotation of human mitochondrial variants reported in the HmtVar database.

**Results:**

The data mining pipeline has produced a list of 1,073 Pubmed IDs (PMIDs) from which the text mining pipeline has retrieved information on 932 human mitochondrial variants regarding experimental validation and clinical features.

**Conclusions:**

The application of the pipeline will contribute to supporting the interpretation of pathogenicity of human mitochondrial variants by facilitating diagnosis to clinicians and researchers faced with this task.

## Graphical abstract

The “data and text mining” pipeline, developed using the programming language R, is capable of extracting information from the literature regarding mtDNA functional studies and related clinical assessments. The application of the pipeline will contribute in supporting the interpretation of pathogenicity of human mitochondrial variants by facilitating diagnosis to clinicians and researchers that approach to this task.

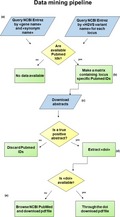

## 1 INTRODUCTION

The human mitochondrial genome is a circular, double‐stranded DNA molecule (mtDNA) of 16,569 bp characterized by peculiar organization and features (Anderson et al., 1981). The mtDNA is transmitted through a maternal inheritance (Giles, Blanc, Cann, & Wallace, 1980); it is characterized by a heteroplasmic nature (Lightowlers, Chinnery, Turnbull, & Howell, 1997), which implies the presence of more than one type of mitochondrial genome within a cell; it shows an extremely economic organization, in which genes lack introns, intergenic sequences are absent or limited to few bases, and protein genes are overlapped (Anderson et al., 1981; Taanman, 1999). The mtDNA codifies for 37 genes, 13 genes for protein, 22 for tRNA, and two for rRNA; in addition, it shows a large noncoding region of 1,133 bp, called D‐loop (displacement loop), characterized by a triple‐strand that is bounded by the genes for tRNA‐Phe and tRNA‐Pro, and related to regulatory activities of the mitochondrial genome (Taanman, 1999). Considering that the mitochondrion is involved in the production of cellular energy through oxidative phosphorylation, mtDNA integrity is heavily exposed to damage by mitochondrial reactive oxygen species (ROS) (Chinnery & Hudson, 2013). Hence, mtDNA is very susceptible to accumulating point variations and other rearrangements that could have negative effects in terms of diseases with a wide range of clinical expressions and phenotypes (Schapira, 2012). However, as widely reported in the literature, a great number of mtDNA variations are fixed in the population, occur with a higher rate than nuclear DNA, and a large number of these changes have no pathogenic significance (Wallace, Brown, & Lott, 1999). In this scenario, the establishment of standard criteria is required to determine the degree of pathogenicity of any mtDNA variant and assign it a clinical role. With this aim, besides the “canonical criteria” described in DiMauro & Shon (DiMauro & Schon, 2001) (Table 1), further approaches have been used to correctly classify mtDNA variants (McFarland, Elson, Taylor, Howell, & Turnbull, 2004), including genetic, biochemical, histochemical, and cellular studies such as trans‐mitochondrial cybrids and single‐fiber cells. In addition, for mitochondrial tRNA variants, the abovementioned types of functional data were improved and associated with a scoring system (Diroma, Lubisco, & Attimonelli, 2016; González‐Vioque, Bornstein, Gallardo, Fernández‐Moreno, & Garesse, 2014; Preste, Vitale, Clima, Gasparre, & Attimonelli, 2019; Yarham et al., 2011) (Table 2), thus allowing the discrimination of pathogenic mutations from neutral polymorphisms. In this context, a pipeline capable of extracting information from the literature regarding mtDNA functional studies and related clinical assessments is proposed here, so as to improve the annotation of the human mtDNA variants as reported in the HmtVar database (https://www.hmtvar.uniba.it/) (Preste et al., 2019).

**Table 1:** Canonical criteria supporting the deleterious role of a novel mutation as reported in DiMauro & Schon, 2002.

Canonical criteria of pathogenicity from DiMauro and Schon (2001 **)**
Mutation must not be a known neutral polymorphism
The base change must affect an evolutionarily conserved and functionally important site
Deleterious mutations are usually heteroplasmic, although a few pathogenic mutations are homoplasmic
The degree of heteroplasmy in different family members ought to be in rough agreement with the severity of symptoms
The single‐fiber PCR as a method that allows the correlation of mutational load and functional abnormality

**Table 2:** The pathogenicity scoring system. The table reports the update of the pathogenicity scoring system according to Yarham criteria (Yarham et al., 2011) and further improved in HmtVar (Preste et al., 2019)

The pathogenicity scoring criteria		Score
Variant described as pathogenic by more than one report	yes	2
	no	0
PhastCons conservation	yes	1
	no	0
PhyloP conservation	yes	1
	no	0
Heteroplasmy evidences	yes	2
	no	0
Segregation of mutation with disease	yes	2
	no	0
Histochemical evidence of mitochondrial disease	yes	2
	no	0
Biochemical defect in OXPHOS complexes I, III, IV	yes	2
	no	0
Pathogenicity evidence in trans‐mitochondrial cybrids or mutant mt‐tRNA steady‐state level studies	yes	5
	no	0
Evidence of mutation segregation with biochemical defect from single‐fiber studies	yes	3
	no	0

Note

Each of the criteria is associated with a weighted score allowing classification of human mitochondrial tRNA variant pathogenicity. The improvements applied in Preste et al., (2019) is focused on PhyloP and PhastCons usage to evaluate the inter‐mammalian site conservation.

## 2 MATERIALS AND METHODS

### 2.1 mtDNA variants dataset

Scripts, written in R (https://www.r-project.org/) and Python (https://www.python.org/), were designed to define the complete list of any possible human mitochondrial DNA variant, defined by the comparison with the revised Cambridge Reference Sequence (rCRS) (Anderson et al., 1981) and reported using the Human Genome Variation Society (HGVS) nomenclature (den Dunnen et al., 2016). The location of each mitochondrial gene in rCRS was retrieved by querying the NCBI‐Nucleotide database (https://www.ncbi.nlm.nih.gov/) using the string “NC_012920.1” related to the Homo Sapiens Mitochondrion Complete Genome. Through the additional resources available at the Phylotree site (http://www.phylotree.org/resources/rCRS_annotated.htm), the reference allele for each rCRS position was used as list of reference alleles**.** Starting from these input data, a complete dataset of all potential 49,726 human mitochondrial variants was generated.

### 2.2 Pipeline framework

**Figure 1:**
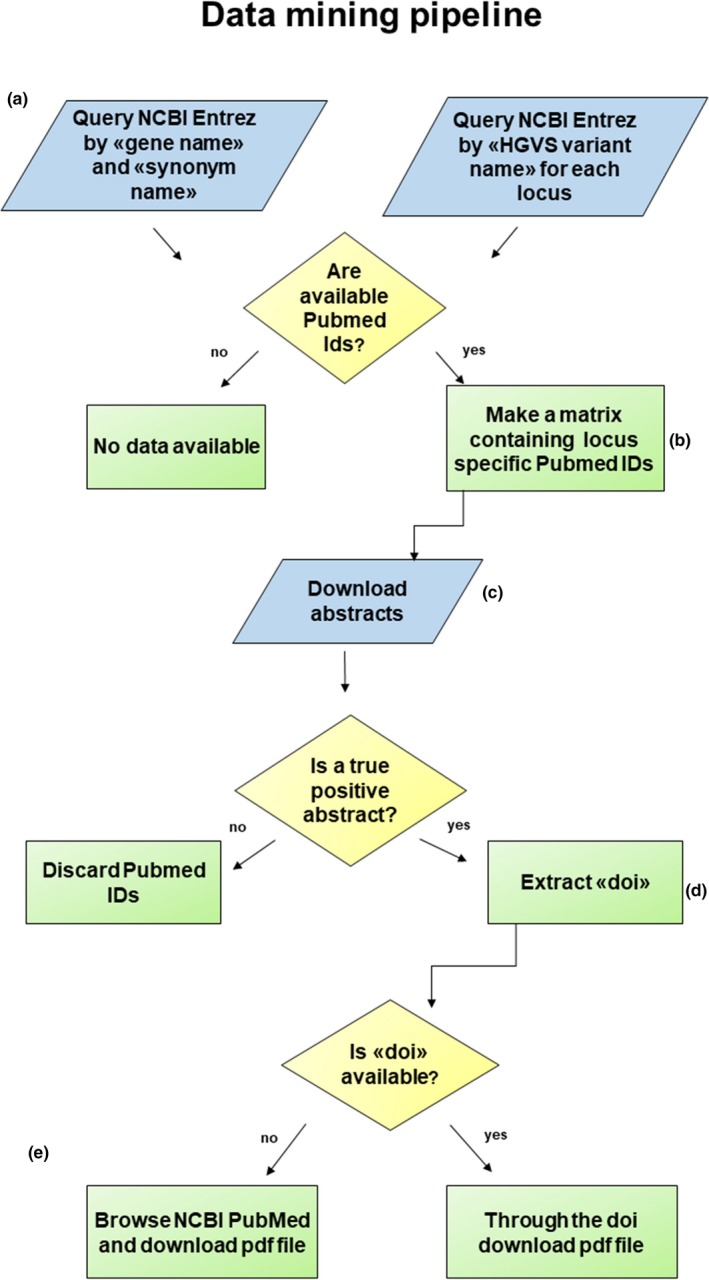
Workflow describing data mining pipeline. For each mtDNA locus, the main steps are: (a) query through the NCBI Entrez system the PubMed database by “gene name or synonym gene name” and “mtDNA variant name” in HGVS format; (b) store the retrieved Pubmed IDs list; (c) download the abstract related to each Pubmed IDs and keep those containing information regarding functional studies and/or variant; (d) for true positive Pubmed IDs, extract the DOI; e) browse NCBI PubMed and download the related PDF articles

**Figure 2:**
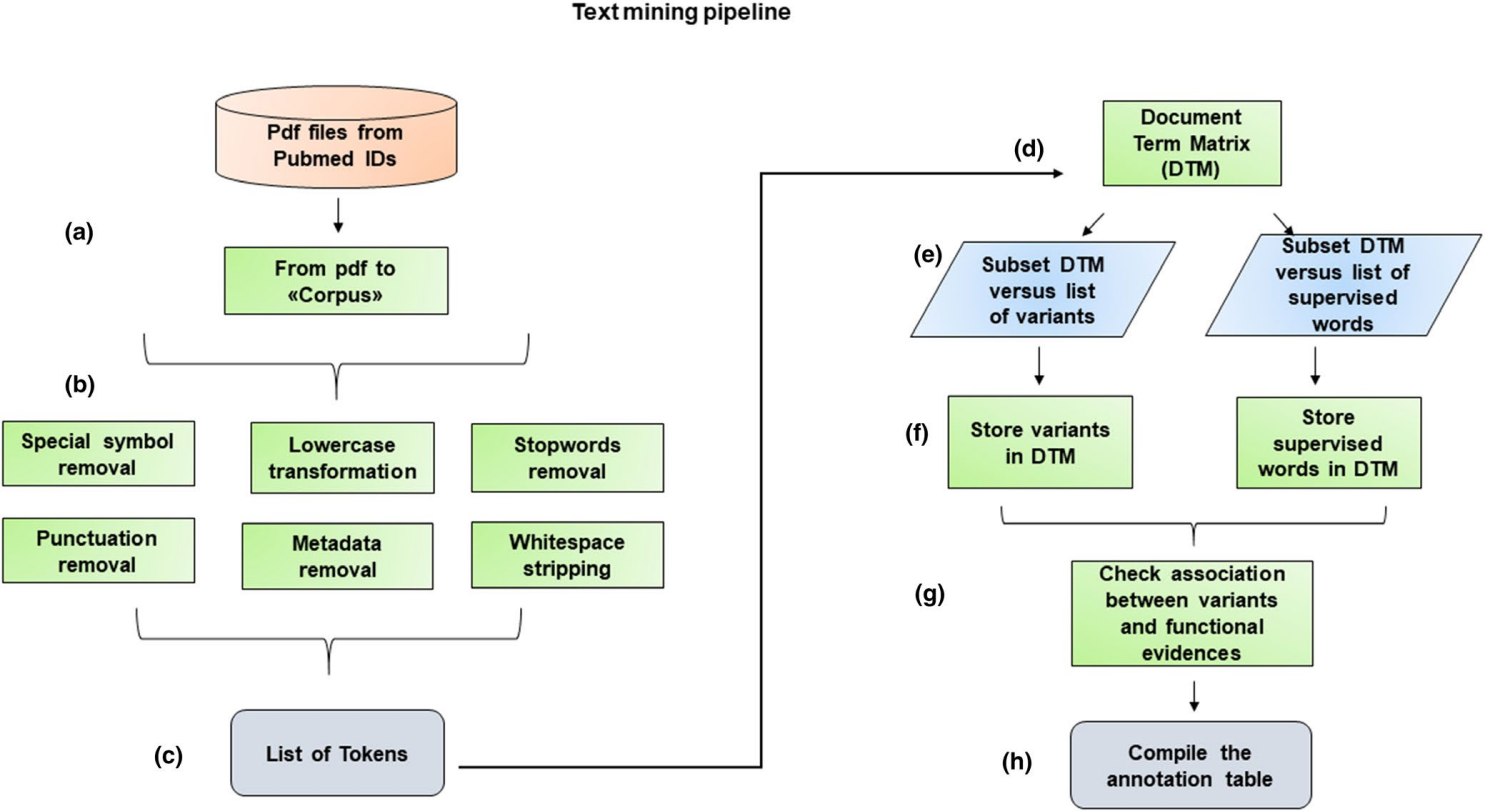
Workflow describing text mining pipeline. The main steps are: (a) transformation of the PDF article text into a “Corpus”; (b) preprocessing of the Corpus; (c) creation of the token list; (d) definition of the Document‐Term Matrix (DTM); (e) subsetting of the DTM versus the list of variant annotated with different token formats and versus the list of supervised words related to functional evidences; (f) creation of the list of mined variants and related annotations; (g) check the association of variants and specific functional evidences; (h) compilation of the annotation tables

The “data and text mining” pipeline framework, written in R, was realized with the purpose of retrieving the information available in literature about any human mitochondrial DNA variant for which functional evidence supporting its clinical status was reported according to the criteria described in Tables 1 and 2. The workflow was structured into two pipelines, “data mining” and “text mining” (Figures 1 and 2).

### 2.3 Data mining pipeline

The “data mining” pipeline is based on the use of “rentrez” and “fulltext” packages. These packages allow the user to retrieve data from the NCBI database “Pubmed” (https://www.ncbi.nlm.nih.gov/pubmed/) using NCBI's E‐utilities (https://www.ncbi.nlm.nih.gov/books/NBK25497/). This pipeline was implemented to use both the “gene name” and the “HGVS variant name” as search terms, in order to obtain a list of Pubmed IDs (PMIDs) for each mitochondrial locus (protein‐coding, tRNA, rRNA, D‐loop). The “gene name” query was based on the name and the synonym name of each specific locus, as reported in the “NC_012920.1” entry, and combined with the terms “human,” “mitochondrial,” and “variant” in order to avoid false results. The “HGVS variant name” query was based on the list of the 49,726 variants annotated according to HGVS nomenclature, that is, m.[Pos][Ref]>[Alt]. Once the pipeline has been applied, the output of these queries results in a unique list of PMIDs. This list is then used as input to automatically download their abstracts. After that, each positive PMID is used to automatically browse the related web page and hence to download it manually. Finally, the selected PDF files are submitted to the text mining pipeline.

### 2.4 Text mining pipeline

The “text mining” pipeline is based on the use of several R packages, among which “tm” and “tidytext” are involved in the main text mining framework concerning data import, Corpus handling and cleaning, preprocessing, and finally the creation of a Document‐Term Matrix (DTM) (Welbers, Atteveldt, & Benoit, 2017). In this pipeline, once the PDF files have been retrieved, they are imported in R in order to be handled for the Corpus implementation. Several preprocessing operations are performed for each Corpus, such as lowercase transformation, whitespaces stripping and special symbols, “stop words”, punctuation, and metadata removal. After these steps, single‐words available in the Corpus are tokenized into the DTM. Once collected and stored, the DTM is further mined by retrieving all possible human mitochondrial variants, whatever their format (Table 3), and any further information about functional evidence according to Yarham's criteria. Starting from these criteria, a list of supervised keywords is generated by browsing the articles in the literature that contain functional data (Table S1). Hence, once both variants and evidence have been stored, the analyst of the process performs tests by checking the context where the selected words were located in the text. Finally, the retrieved data are used to annotate the human mitochondrial DNA variants with functional information regarding experimental validation.

**Table 3:** Token Formats. The table lists, in addition to the standard HGVS nomenclature, the most used formats by which mitochondrial variants are reported in the literature

token_format
m.[POS][REF]>[ALT]
m.[POS][REF][ALT]
[REF][POS][ALT]
[POS][ALT]
m.[POS][REF]

## 3 RESULTS

### 3.1 Data mining results

**Figure 3:**
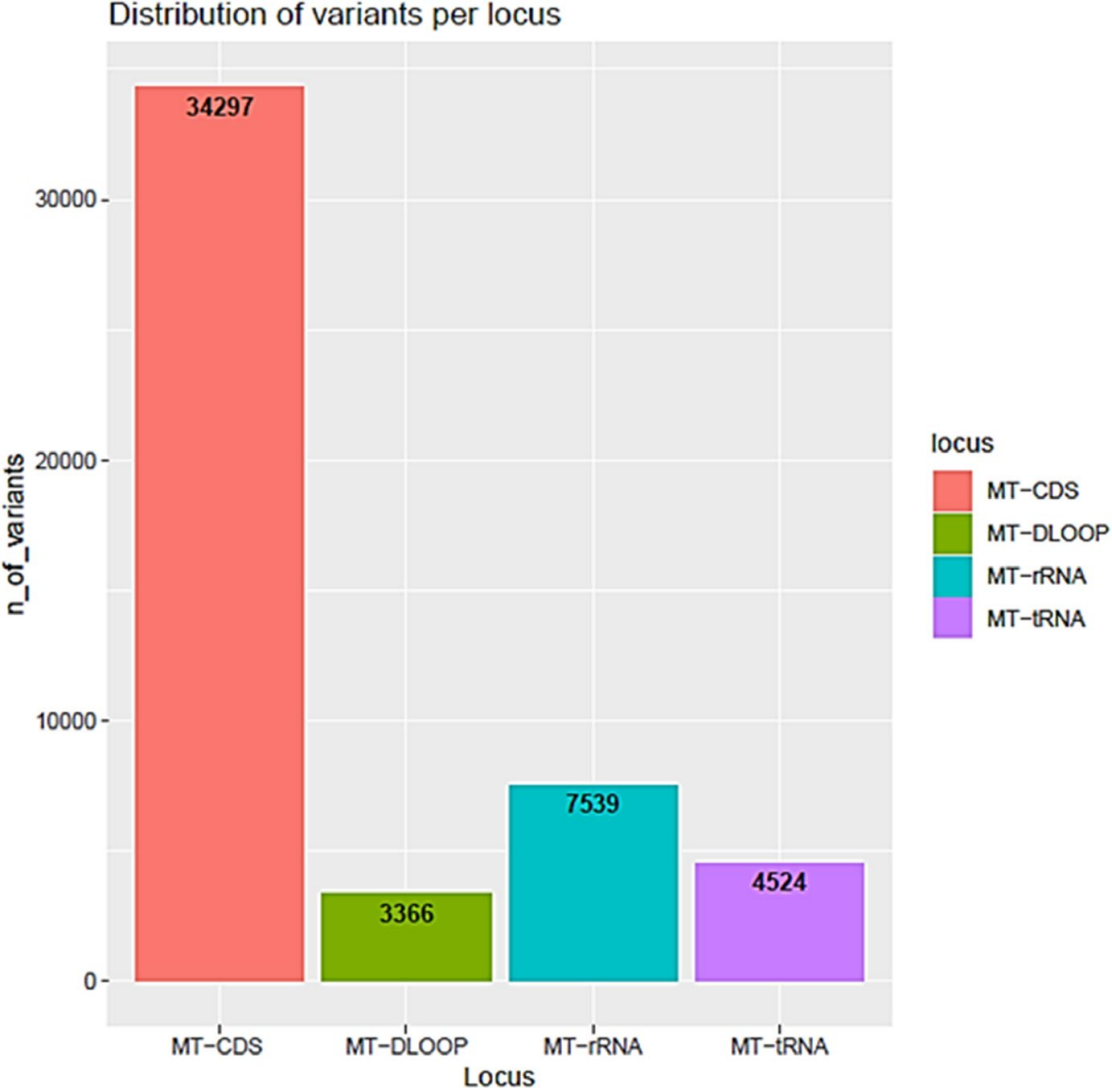
Distribution of variants per locus. The barplot shows the number of total potential human mitochondrial single‐nucleotide variants for each mitochondrial locus (mt‐CDS:34297, mt‐DLOOP:3366, mt‐rRNA:7539, mt‐tRNA:4524)

The analysis of the 49,726 human mitochondrial variants is implemented for each locus. The distribution of any possible variants, reported in Figure 3, refers to single‐nucleotide substitutions. By applying the data mining pipeline on any human mitochondrial locus, a list of PMIDs is produced. In the application of the pipeline in December 2018, 642 PMIDs for protein‐coding, 259 for tRNA, 96 for rRNA, and 76 for D‐loop region were produced.

### 3.2 Text mining results

Starting from the retrieved PMIDs, through the application of the text mining pipeline, 932 human mitochondrial variants with relevant information were retrieved. The information regarding functional studies and their association with diseases and phenotypes as well as conservation data was extracted from the literature and annotated in HmtVar (Preste et al., 2019). It is worth mentioning that for both the tRNA and protein‐coding variants, within HmtVar, a scoring system is implemented allowing each variant to be assigned to a specific tier of pathogenicity (Preste et al., 2019). For the tRNA variants, this feature is implemented by taking into account the Yarham scoring system (Yarham et al., 2011) and hence the information extracted through the text mining pipeline. For the protein‐coding variants, the scoring system is estimated according to Santorsola et al. (2016) and derived from the weighted mean of six pathogenicity predictors. Hence, the functional information here extracted is annotated in HmtVar (Preste et al., 2019) as ancillary textual data.

### 3.3 tRNA variants

Despite the fact that for tRNAs the annotation of the clinical significance of the variants was previously made and then reported in HmtVar (Preste et al., 2019), the application of the pipeline to tRNA variants allowed the updating of both already annotated and un‐annotated tRNA variants for a total of 217 tRNA variants (Table S2) and hence of the tRNA disease scores as estimated from the application of Yarham's pathogenicity scoring system (Diroma et al., 2016; Preste et al., 2019; Yarham et al., 2011).

### 3.4 Protein‐coding variants

The text mining pipeline retrieved 465 variants mapping on protein‐coding genes associated with information about experimental validations and clinical features (Table S3). Considering the fact that for protein‐coding variants a validated method to classify variants in tiers (Preste et al., 2019) is available and adopted in HmtVar, with the aim to offering a widespread vision about available functional data, the retrieved data are reported as ancillary information in HmtVar.

### 3.5 D‐loop and rRNA variants

After applying the text mining pipeline, a total number of 162 and 88 variants were extracted for D‐loop and rRNA loci, respectively. For these regions, no methods to classify variants in a specific tier of pathogenicity are available. However, we have contributed to identifying variants that are surely known as being associated with a disease and to creating a compendium of functional data about them (Tables S4 and S5).

### 3.6 Quality of text mining pipeline

To evaluate the performance of the pipeline, we have compared the annotation status of the 932 variants with that reported at the time of the analysis in other databases, such as Mitomap (Brandon et al., 2005; Lott et al., 2013), Clinvar (Landrum et al., 2014), and OMIM (Hamosh, 2002) (Table 4). The results show that the percentages of additional information due to the pipeline amount to 60.41%, 82.08%, and 87.02%, respectively. For example, the variants m.15990C > A and m.7480T > C, located in tRNA loci, are not annotated in Mitomap; pipeline results, however, report various types of functional evidence regarding their involvement in myopathy. Moreover, for the protein‐coding variants m.8839G > C and m.15132T > C, we have mined information that clarifies the involvement of these variants in NARP syndrome and cardiomyopathy. For rRNA and D‐loop variants, m.2236T > C and m.16362T > C, the common functional evidence retrievable was segregation data that suggest their role in cardiomyopathy and different types of cancer, respectively. However, considering that segregation evidence is informative about a possible genotype–phenotype relationship, but not strong evidence of pathogenicity of a given variant, this stand‐alone information suggests only a likely role of these variants in these disorders. Moreover, the additional information retrieved by the pipeline allowed the quality of annotations already available on HmtVar, to be increased, focusing on the experimental and clinical data as compared to other databases.

**Table 4:** Comparison with Mitomap, Clinvar, and OMIM databases

Locus_type	Mitomap_variant	Pipeline_variant	Shared_variant	only_Mitomap	only_Pipeline
Protein‐coding	337	465	185	152	280
D‐loop	23	162	11	12	151
rRNA	56	88	28	56	59
tRNA	283	217	144	139	73
total	699	**932**	368	359	**563**

Locus_type	Clinvar_variant	Pipeline_variant	shared_variant	only_Clinvar	only_Pipeline
Protein‐coding	363	465	99	264	366
D‐loop	0	162	0	0	162
rRNA	66	88	7	59	81
tRNA	132	217	61	71	156
total	561	**932**	167	394	**765**

Locus_type	OMIM_variant	Pipeline_variant	shared_variant	only_OMIM	only_Pipeline
Protein‐coding	103	465	60	43	405
D‐loop	1	162	0	1	162
rRNA	8	88	6	2	82
tRNA	85	217	55	30	162
total	197	**932**	121	76	**811**

Note

The table shows the number of variants for each locus annotated in Mitomap, Clinvar, OMIM, and the ones mined by the data and text mining pipeline. In addition, the number of variants common between the databases and the pipeline is reported in the shared_variant column; the only_Mitomap/Clinvar/OMIM column reports the number of variants stored in these database that the pipeline does not able to extract; the column only_Pipeline contains the number of variants for which annotation about functional data and diseases are available that are not present in other databases. The full annotation and information about the human mitochondrial variants are available on the HmtVar database where all these data are reported.

Bold indicates the resultant data extracted from the pipeline.

## 4 CONCLUSIONS

The classification of human mitochondrial variants is pivotal for clinicians and researchers to understand and clarify the pathogenicity or neutrality of a certain variation. Even if there are reports in the literature of different research groups which have approached this task (DiMauro & Schon, 2001; Yarham et al., 2011) and have proposed golden standard criteria to use for interpretation of variants, a system able to locate the information related to these criteria has not been previously developed. Hence, nowadays the user has to search for functional and clinical information without automatic support. In this context, our contribution consists of the development of a data and text mining pipeline able to retrieve human mitochondrial variants from the literature and associate experimental evidences and clinical information to them in order to confirm or exclude their pathogenic role. Hence, our goal was based on the assessment of a compendium of data that allow clinicians and researchers to have an overview about features of human mitochondrial variants. Obviously, these data should be updated periodically, in order to constantly extract new information that could enrich the data already available in HmtVar (Preste et al., 2019). Moreover, the evaluation of these criteria has to be considered as a robust proof of pathogenicity of variants not in a stand‐alone manner but considering a combination of evidence that supports the deleterious effect of a given variant. Our hope is to contribute to supporting the interpretation of pathogenicity of human mitochondrial variants by facilitating diagnosis for clinicians and researchers faced with this task.

## CONFLICT OF INTEREST

No potential conflict of interest was reported by the authors.

## AUTHORS CONTRIBUTION

All the authors contributed equally to the design and implementation of the research, to the analysis of results, and to the writing of the manuscript.
